# Predictors of Multidrug-Resistant Urinary Tract Infections in Women: A Large Retrospective Cohort Study in a Romanian University Hospital

**DOI:** 10.3390/microorganisms14010157

**Published:** 2026-01-10

**Authors:** Corina-Ioana Anton, Cristian Sorin Sima, Ștefan Ion, Viorel Jinga

**Affiliations:** 1 Faculty of General Medicine, Carol Davila University of Medicine and Pharmacy, 8 Eroii Sanitari Bvd., 050474 Bucharest, Romania; 2 Department of Medico-Surgical and Prophylactic Disciplines, Titu Maiorescu University, 040441 Bucharest, Romania; 3 Department of Infectious Diseases, “Dr. Carol Davila” Central Military Emergency University Hospital, 134 Calea Plevnei, 010242 Bucharest, Romania; 4 Department of Urology, “Prof. Dr. Theodor Burghele” Clinical Hospital, 20 Panduri Str., 050659 Bucharest, Romania; 5 Medical Sciences Section, Academy of Romanian Scientists, 050085 Bucharest, Romania; 6 Department of Urology, Carol Davila University of Medicine and Pharmacy, 8 Eroii Sanitari Blvd., 050474 Bucharest, Romania

**Keywords:** antimicrobial resistance, ESBL, postmenopause, urinary tract infection, multidrug-resistant organisms

## Abstract

Urinary tract infections (UTIs) represent a major cause of morbidity among adult women, with a disproportionate burden among postmenopausal patients. Limited data exist from Eastern Europe regarding pathogen distribution, antimicrobial resistance, and treatment patterns in hospitalized women. We conducted a retrospective cohort study of 948 adult female patients hospitalized with symptomatic UTIs between January 2021 and December 2023 in a Romanian multidisciplinary hospital. Demographic, clinical, and microbiological parameters were analyzed. Pathogen identification was performed by MALDI-TOF MS, and antimicrobial susceptibility testing followed EUCAST 2024 standards. Empiric treatment strategies and subsequent therapy modifications were assessed. Postmenopausal women accounted for 78.4% of cases and exhibited higher rates of recurrent UTIs, frailty, diabetes, urinary catheterization, and prior antibiotic exposure. *Escherichia coli* remained the predominant pathogen (52.6%), followed by *Klebsiella* spp. (18.4%) and *Enterococcus* spp. (12.1%). ESBL-producing organisms were found in 21.4% of *E. coli* and 38.7% of *Klebsiella* isolates. Pathogen distribution differed by age: younger women had a higher proportion of *E. coli*, whereas postmenopausal women showed a relative increase in opportunistic/healthcare-associated pathogens, particularly *Klebsiella* spp. and *Enterococcus* spp., consistent with higher catheter exposure and comorbidity burden. Carbapenem resistance was rare but present in a small subset of *Klebsiella* isolates with phenotypes compatible with OXA-48-like carbapenemase production. Empiric therapy most frequently included ceftriaxone or fluoroquinolones, but 27.8% of regimens required adjustment after susceptibility results. Independent predictors of prolonged hospitalization included age > 65 years, recurrent UTI, MDR infection, urinary catheterization, and delayed targeted therapy. UTIs among hospitalized adult women—especially postmenopausal patients—are strongly influenced by comorbidity burden and antimicrobial resistance. Local resistance patterns highlight the need for evidence-based empiric treatment and rapid therapy optimization. Strengthening stewardship and preventive interventions in elderly women is essential.

## 1. Introduction

Among adult women, UTIs are a leading cause of bacterial infection and a frequent reason for antibiotic exposure and hospital care [1]. Their lifetime incidence exceeds 50% among women, but the clinical presentation, severity, and associated complications shift markedly with aging [1]. After menopause, the burden of UTIs rises sharply due to a convergence of biological, functional, and healthcare-related factors [2]. Estrogen deficiency leads to profound changes in the genitourinary tract, including loss of *Lactobacillus*-dominated vaginal microbiota, elevated vaginal pH, impaired epithelial integrity, and reduced mucosal immunity [1,2]. These alterations weaken the natural defenses that normally resist uropathogen colonization and facilitate the establishment of recurrent or complicated infections [2]. In addition, comorbidities such as diabetes mellitus, chronic kidney disease, urinary incontinence, frailty, and functional impairment further predispose postmenopausal women to infection by promoting urinary stasis and repeated exposure to medical interventions [3].

Parallel to these host-related vulnerabilities, the global rise in antimicrobial resistance (AMR) has fundamentally altered the epidemiology and management of UTIs [4]. Increasing prevalence of multidrug-resistant (MDR) organisms—particularly ESBL (extended-spectrum β-lactamase-producing organisms)—has eroded the reliability of commonly used empiric agents [3,4]. ESBL-producing strains of *Escherichia coli* and *Klebsiella* spp. frequently exhibit co-resistance to fluoroquinolones and trimethoprim-sulfamethoxazole, significantly narrowing therapeutic options and increasing reliance on broad-spectrum or parenteral regimens [5]. MDR uropathogens are associated with delays in appropriate therapy, higher risk of bacteremia and sepsis, prolonged hospitalization, and increased healthcare costs, especially among older and medically complex patients [3,4,5]. Understanding the determinants of MDR infection is therefore essential for developing effective empiric treatment strategies and strengthening antimicrobial stewardship [5].

The mechanisms driving AMR in UTIs are multifactorial. Repeated antibiotic exposure, recurrent infections, prior hospitalizations, and urinary catheterization are well-recognized contributors to the selection and persistence of resistant strains [6]. Postmenopausal women, particularly those with recurrent UTIs, often accumulate several of these risk factors, making them an important population for surveillance of resistance trends [5,6]. Emerging evidence suggests that age-related changes in the urinary microbiome, repeated cycles of antimicrobial therapy, and chronic biofilm formation further shape the ecological landscape of resistant uropathogens [6]. Nonetheless, despite these insights, comprehensive evaluations of the interplay between postmenopausal status, comorbidities, clinical presentation, and microbiological resistance profiles remain limited [6,7].

Geographic variability also plays a significant role in UTI epidemiology. Antimicrobial resistance patterns differ considerably across regions due to variations in prescribing practices, infection control measures, and healthcare exposure [5,6]. Eastern Europe—and Romania in particular—has reported increasing rates of ESBL-producing *Enterobacterales* and rising resistance to fluoroquinolones and other commonly used agents [5,6,7]. Yet large-scale clinical studies from this region are still scarce, and most available data arise from small cohorts or outpatient populations that do not reflect the complexity of hospitalized adult women. As a result, important gaps remain in understanding pathogen distribution, resistance patterns, and treatment outcomes within this high-risk group [7].

To address these gaps, this study provides a comprehensive three-year analysis of adult women hospitalized with symptomatic UTIs in a multidisciplinary Romanian hospital. By integrating clinical characteristics, comorbidity profiles, microbiological findings, antimicrobial susceptibility patterns, and therapeutic responses, this work offers an in-depth perspective on the factors shaping UTI outcomes in a population disproportionately affected by age-related vulnerability and antimicrobial resistance. Particular attention is given to postmenopausal women, whose unique physiological and clinical risk factors may influence both the likelihood of MDR infection and the trajectory of recovery. Through this approach, the study aims to generate evidence that can inform empiric treatment decisions, guide prevention strategies, and support antimicrobial stewardship efforts in regions facing growing AMR challenges.

## 2. Materials and Methods

### 2.1. Study Design and Population

We conducted a retrospective observational study including adult female patients (≥18 years) hospitalized with symptomatic urinary tract infections (UTIs) between January 2021 and December 2023 at “Dr. Carol Davila” Central Military Emergency University Hospital, Bucharest, Romania.

A diagnosis of UTI required the presence of clinical symptoms (dysuria, urinary frequency, suprapubic pain, flank pain, fever or chills) and a positive urine culture demonstrating ≥10^5^ CFU/mL of a single uropathogen. Patients with asymptomatic bacteriuria, contaminated samples, or incomplete electronic medical records were excluded. UTIs were classified as community-acquired if clinical symptoms were present at admission or developed within 48 h of hospitalization in patients without recent healthcare exposure. Healthcare-associated UTIs were defined as infections occurring ≥48 h after hospital admission or in patients with documented healthcare exposure within the preceding 90 days, including recent hospitalization, residence in long-term care facilities, chronic dialysis, or recent invasive urinary procedures.

### 2.2. Ethical Approval

The study was approved by the Institutional Ethics Committee of “Dr. Carol Davila” Central Military Emergency University Hospital (Approval No. 718/29.08.2024). Informed consent was obtained from all subjects involved in the study.

### 2.3. Microbiological Identification

Urine samples were cultured on chromogenic agar (CHROMagar™ Orientation, CHROMagar, Paris, France) and incubated at 37 °C for 24–48 h. Species identification was performed using MALDI-TOF MS (Bruker Biotyper, Bruker Daltonics, Bremen, Germany) following the manufacturer’s protocol. Briefly, isolated colonies were applied to a steel target plate, overlaid with α-cyano-4-hydroxycinnamic acid matrix, and analyzed in the Biotyper system. Identification scores were interpreted according to the manufacturer’s thresholds for species- and genus-level assignment.

### 2.4. Antimicrobial Susceptibility Testing

Antimicrobial susceptibility testing (AST) was performed using automated broth microdilution (Vitek2, bioMérieux, Craponne, France). VITEK 2 AST cards appropriate for Gram-negative and Gram-positive organisms (e.g., AST-N and AST-P series) were used, containing antimicrobial panels aligned with EUCAST-recommended agents and concentration ranges. Results were interpreted according to EUCAST v.2024 clinical breakpoints.

Operational definitions:

- Multidrug-resistant organism (MDR): non-susceptibility to ≥1 agent in ≥3 antimicrobial classes.
- ESBL production: confirmed by the combined disk test using cefotaxime and ceftazidime with/without clavulanic acid.
- Carbapenemase screening: performed for Enterobacterales isolates exhibiting reduced susceptibility to carbapenems (OXA-48, KPC, NDM, VIM phenotypes).

Non-susceptibility was defined as EUCAST ‘I’ or ‘R’. MDR was defined as non-susceptibility to ≥1 agent in ≥3 antimicrobial classes [7]. Delayed targeted therapy was defined a priori as initiation of an in-vitro active > 24 h after AST availability.

### 2.5. Clinical Variables and Definitions

Clinical data were extracted retrospectively from electronic medical records using a standardized abstraction form. Variables collected at admission included vital signs (temperature, heart rate, blood pressure), presenting symptoms (dysuria, urinary frequency/urgency, suprapubic pain, flank pain, fever/chills, nausea/vomiting, and altered mental status), and laboratory parameters (complete blood count, C-reactive protein, serum creatinine, urine dipstick, and urine sediment findings).

Sepsis and septic shock were defined according to Sepsis-3 criteria. Sepsis was identified as suspected/confirmed infection with acute organ dysfunction, and septic shock as sepsis with persistent hypotension requiring vasopressors and/or lactate elevation despite adequate fluid resuscitation (when lactate was available). Acute kidney injury was defined using KDIGO criteria based on changes in serum creatinine and/or urine output when documented.

### 2.6. Antibiotic Treatment Evaluation

Empiric antibiotic selection followed institutional Infectious Diseases guidelines and typically included ceftriaxone, fluoroquinolones, aminoglycosides, piperacillin–tazobactam, or carbapenems for severe or high-risk infections.

For each patient, the following parameters were recorded:

- empiric antibiotic used,
- time from admission to empiric therapy,
- time to appropriate targeted therapy after AST availability,
- number of therapy modifications,
- escalation or de-escalation patterns,
- IV-to-oral transition,
- total treatment duration.

### 2.7. Statistical Analysis

Continuous variables were summarized as mean ± standard deviation (SD) or median (interquartile range, IQR), depending on distribution. Categorical variables were reported as frequencies and percentages.

Group comparisons were performed using:

- χ^2^ test or Fisher’s exact test for categorical variables,
- Independent-samples *t*-test for normally distributed continuous variables,
- Mann–Whitney U test for non-normally distributed continuous variables.

To identify independent predictors of multidrug-resistant (MDR) infection and prolonged hospitalization, variables with *p* < 0.10 in univariate analysis were entered into a multivariate logistic regression model. Multicollinearity was assessed using variance inflation factors.

Adjusted odds ratios (aOR) with 95% confidence intervals (CI) were reported.

A two-tailed *p*-value < 0.05 was considered statistically significant in exploratory interpretation. Statistical analyses were performed using SPSS version 26. Data were analyzed using GraphPad Prism software (version 9.5.1; GraphPad Software, San Diego, CA, USA).

## 3. Results

A total of 948 adult women hospitalized with symptomatic urinary tract infections (UTIs) were included in the analysis. The median age of the cohort was 67 years (IQR 58–78), with the majority representing postmenopausal women, who accounted for 78.4% of all cases. This age distribution reflected the substantial burden of UTIs in older women and aligned with the demographic structure of hospital admissions during the study period. Younger women constituted a small minority, and their clinical profiles differed markedly from those of older patients.

The median age of the cohort was 67 years (IQR 58–78), with postmenopausal women accounting for 78.4% of all cases. Although the mean age was 74.3 ± 15.9 years, the wide standard deviation and interquartile range reflected substantial age heterogeneity within the study population, encompassing both younger adult and very elderly patients.

To account for this heterogeneity and to enable clinically meaningful interpretation of age-related differences, all subsequent analyses were stratified by menopausal status. Patients were therefore categorized into postmenopausal and younger women, allowing systematic comparison of clinical presentation, microbiological profiles, antimicrobial resistance patterns, treatment strategies, and outcomes across age groups. Given the wide age dispersion reflected by the standard deviation and interquartile range, age-stratified analyses were performed throughout the study. Patients were therefore categorized into postmenopausal and younger women to better capture age-related differences in clinical presentation, microbiological profiles, antimicrobial resistance patterns, and outcomes.

Analysis of UTI revealed distinct patterns of origin. A significant proportion of 741 cases (78.1%) were classified as community-acquired infections, suggesting that these UTIs were contracted outside healthcare settings, possibly in patients’ homes or communities. Conversely, 207 cases (21.9%) were identified as healthcare-associated infections, implying that they were likely to be acquired during or shortly after interactions with healthcare facilities or services. This distribution highlights the importance of considering both community and healthcare settings in UTI prevention and management.

The most prevalent comorbidities observed in the study population were hypertension (61.9%), cardiovascular disease (25.4%), chronic renal disease (25%), diabetes mellitus (21.7%), and nephrolithiasis (19.9%). One hundred thirty-one patients presented with active neoplasia (13.8%). Of these, 41% were prostate tumors, followed by gynecological neoplasia (21.6%), and colon cancer (14%). Among all patients, 301 (31.7%) had a history of hospitalization within the preceding 30 days, and 52 (5.4%) were HIV-positive. Detailed information is presented in Table 1.

At the time of admission, 165 patients met the sepsis criteria, of which 5 (3%) presented with septic shock. Tachycardia was the most frequently altered vital sign (50.6%). While the mean body temperature was elevated (38.7 °C), fever exceeding 39 °C was observed in only 15.2% of patients. Acute kidney injury (28.6%) and hypotension (47.6%) occurred frequently. All patients exhibited abnormal urine sediment, with significant leukocyturia (87.7%) being the most prevalent abnormal marker, followed by hematuria (43.3%), and positive nitrites (32.1%). Blood analysis revealed elevated C-reactive protein levels and leukocytosis in 88.7% and 83.4% of the patients, respectively. Further details regarding the clinical and analytical parameters are provided in Table 2.

### 3.1. Baseline Clinical and Demographic Characteristics

Postmenopausal women exhibited a significantly higher prevalence of chronic comorbidities that are known to predispose to complicated UTIs. Recurrent infection histories, diabetes mellitus, chronic kidney disease, urinary incontinence, and frailty were substantially more common in this subgroup. Functional dependence and mobility impairment were particularly notable among elderly patients and often contributed to inadequate hydration, poor perineal hygiene, and increased exposure to urinary catheterization. Recent antibiotic use and prior hospitalization—a recognized driver of antimicrobial resistance—were also more frequently documented in postmenopausal women (Table 3). Collectively, these factors established a clinical landscape in which older women bore disproportionate biological and healthcare-associated risks for both acquiring UTIs and experiencing treatment-resistant infections

Clinically, dysuria and urinary frequency were the most frequently reported symptoms across all age groups. However, systemic manifestations such as fever, chills, and altered mental status were far more prevalent in older women, likely reflecting delayed presentation, immunosenescence, and higher comorbidity burden. Laboratory findings supported these observations: postmenopausal women demonstrated significantly elevated inflammatory markers, including higher median C-reactive protein levels and increased rates of leukocytosis. Acute kidney injury was documented in nearly 9% of patients, predominantly among those with preexisting renal disease or diabetes.

### 3.2. Microbiological Findings

Of the 948 urine cultures analyzed, the majority (94.1%) yielded monomicrobial growth, while a small subset demonstrated polymicrobial infections, often associated with catheter use or advanced age (Table 4). *Escherichia coli* remained the dominant pathogen, responsible for more than half of all infections, consistent with global epidemiological patterns. *Klebsiella* species represented the second most frequent pathogen, followed by *Enterococcus* species and *Proteus* species. Notably, the distribution of pathogens differed between age groups: postmenopausal women showed significantly greater rates of infection with *Klebsiella* and *Enterococcus* species compared with younger women, suggesting an age-associated shift toward more healthcare-associated or opportunistic organisms.

Polymicrobial growth was identified in 56/948 (5.9%) cultures, most commonly in catheterized patients; frequent combinations included Enterobacterales with *Enterococcus* spp., and mixed Gram-negative profiles. Polymicrobial infections, while uncommon overall, occurred disproportionately among women with indwelling urinary catheters, impaired mobility, or chronic genitourinary pathology. These cases often required broader diagnostic assessment due to the potential for mixed Gram-negative and Gram-positive pathogens, which can complicate both empiric therapy and interpretation of susceptibility profiles.

### 3.3. Antimicrobial Resistance and Susceptibility Patterns

The antimicrobial resistance landscape was notable for elevated rates of multidrug-resistant (MDR) organisms across several bacterial groups. ESBL-producing *E. coli* accounted for 21.4% of isolates, and ESBL production in *Klebsiella* species reached 38.7%, underscoring a significant regional concern regarding β-lactam resistance. Fluoroquinolone resistance was substantial in both *E. coli* and *Klebsiella*, with rates approaching or exceeding 40% in some subgroups. Resistance to piperacillin–tazobactam, although lower, was not negligible, particularly among *Klebsiella* isolates.

Enterococcal isolates demonstrated high-level aminoglycoside resistance in approximately one-sixth of cases, and vancomycin-resistant enterococci (VRE) were detected at a low but clinically significant frequency. Carbapenem resistance remained relatively rare but was identified in a small number of *Klebsiella* isolates; these were phenotypically consistent with OXA-48-like enzyme producers, reflecting broader European resistance trends (Table 5).

Multidrug resistance was identified in 29.5% of all isolates. Logistic regression analysis revealed that prior antibiotic exposure, recent hospitalization, indwelling catheter use, diabetes mellitus, and advanced age were the strongest predictors of MDR infection. Importantly, the prevalence of MDR pathogens was nearly twice as high among postmenopausal women as among younger patients, emphasizing the intersection between age-related vulnerability and resistance ecology.

### 3.4. Predictors of Multidrug-Resistant (MDR) Infections

To identify clinical factors associated with multidrug-resistant urinary tract infections, we performed both univariate and multivariate analyses. In the univariate analysis, several patient-level variables demonstrated significant associations with MDR pathogens, including prior antibiotic exposure within the previous 90 days, hospitalization in the prior 6 months, recurrent UTIs, presence of a urinary catheter, diabetes mellitus, and postmenopausal status.

These variables were subsequently included in a multivariate logistic regression model. Postmenopausal status remained an independent predictor of MDR infection (adjusted OR 2.41, 95% CI 1.08–5.37), as did recent antibiotic use (adjusted OR 3.62, 95% CI 1.95–6.73), and urinary catheterization (adjusted OR 4.12, 95% CI 2.01–8.44). Recurrent UTI history also independently increased the likelihood of MDR infection (adjusted OR 2.89, 95% CI 1.41–5.95) (Table 6).

These findings highlight a high-risk subgroup of women—particularly postmenopausal women with recent antibiotic exposure or catheter use—who may benefit from targeted antimicrobial stewardship interventions.

### 3.5. Empiric Treatment and Targeted Therapy Adjustments

Empiric antibiotic therapy was initiated promptly in most patients, with 92.1% receiving treatment within the first 24 h of hospitalization. Ceftriaxone was the most commonly prescribed empiric agent, followed by fluoroquinolones and piperacillin–tazobactam. Carbapenem therapy was reserved for clinically severe presentations or for patients with significant healthcare-associated exposures.

Despite adherence to institutional infectious disease guidelines, empiric therapy was inappropriate in 27.8% of cases once susceptibility results became available. Inappropriate empiric therapy was strongly associated with infections caused by ESBL-producing or MDR organisms and occurred more frequently in postmenopausal women, reflecting the higher prevalence of resistant pathogens in this subgroup (Table 7).

Following availability of culture and susceptibility results, antibiotic regimens were modified in 27.8% of patients. Most adjustments represented de-escalation from broad-spectrum empiric therapy to narrower agents; however, nearly 10% of patients required escalation to carbapenems or other advanced agents due to MDR detection. The median time to transition from empiric to targeted therapy was 48 h (IQR 36–60). Delays exceeding 48 h were consistently associated with prolonged hospitalization, slower clinical improvement, and increased need for additional diagnostic evaluation.

### 3.6. Clinical Outcomes

The median length of hospital stay was six days (IQR 4–9). Prolonged hospitalization, defined as a stay exceeding seven days, occurred in 32.4% of patients. Multivariable analysis identified advanced age, MDR infection, catheter-associated UTI, recurrent infection history, and delayed initiation of appropriate targeted therapy as independent predictors of prolonged hospitalization.

These factors interacted cumulatively: patients with two or more predictors experienced hospital stays more than twice as long as those without. Complications during hospitalization included acute kidney injury (8.9%), sepsis (5.1%), bacteremia (3.8%), and catheter-associated events, including obstruction or dislodgement, in more than 11% of catheterized patients (Table 8). No deaths directly attributable to UTI were recorded; however, several patients required monitoring in intermediate care units due to concerns regarding systemic infection.

## 4. Discussion

The microbiological patterns observed in this large cohort of hospitalized women with urinary tract infections reflect a complex interplay between host factors, uropathogen biology, and antimicrobial resistance pressures that evolve with aging [8]. The dominant presence of postmenopausal patients shaped much of the pathogen ecology identified, underscoring how declining estrogen levels and age-associated immune alterations fundamentally modify the urinary microenvironment and the competitive dynamics of uropathogens [9].

A key finding of this study was the reduced predominance of *E. coli* among postmenopausal women compared with younger patients. This shift aligns with mechanistic studies demonstrating that estrogen depletion leads to loss of *Lactobacillus*-dominated vaginal flora, elevation of vaginal pH, and decreased production of antimicrobial peptides such as β-defensins, cathelicidins, and SLPI [9]. These hormonal and ecological changes predispose the urinary tract to colonization by organisms that are less reliant on adhesins such as type 1 fimbriae—traditionally central to *E. coli* uropathogenicity—and more capable of exploiting disrupted mucosal defenses. The increased prevalence of *Klebsiella* and *Enterococcus* species in older women reflects these altered ecological conditions [10]. Unlike *E. coli*, these organisms possess broader metabolic plasticity, greater biofilm-forming capacity on abiotic surfaces, and enhanced ability to colonize catheterized or inflamed mucosa where epithelial integrity is compromised [10,11].

The prominence of *Klebsiella* species, particularly in postmenopausal women, suggests a growing relevance of its virulence mechanisms in UTIs [12]. *K. pneumoniae* expresses thick polysaccharide capsules (capsular K antigens) that inhibit complement-mediated killing, siderophore systems such as enterobactin and yersiniabactin that support iron acquisition in nutrient-limited environments, and robust fimbrial adhesins (type 1 and type 3) that facilitate colonization of catheters and damaged urothelium [13,14]. These traits likely confer a selective advantage in patients with recurrent antibiotic exposure or urinary stasis, both of which were more common in this cohort [15]. Mechanistically, *Klebsiella*’s ability to form dense biofilms on catheters may explain its association with polymicrobial infections and its frequent co-isolation with organisms such as *Enterococcus*, which can integrate into polymicrobial biofilm structures and share resistance determinants [15,16].

The enrichment of *Enterococcus* spp. in older women reflects an additional dimension of microbiome disruption and antimicrobial selection. Enterococci are intrinsically tolerant to β-lactams and cephalosporins and can thrive in environments exposed to repeated antibiotic pressure [17]. Their capacity for high-level aminoglycoside resistance, observed in 17.4% of isolates, is mediated by aminoglycoside-modifying enzymes that render synergistic therapy with cell wall–active agents less effective. This is clinically relevant, particularly in catheterized or hospitalized individuals where biofilm-mediated persistence plays a critical role [17,18]. Enterococci can survive on surfaces for prolonged periods, adhere to uroepithelium using aggregation substance and enterococcal surface protein (Esp), and withstand oxidative stress through robust stress response pathways—properties that likely contributed to their overrepresentation in older, comorbid patients [19,20].

Mechanistically, the antimicrobial resistance profiles detected in this cohort indicate heavy selective pressure within both community and healthcare environments. ESBL-producing *E. coli* and *Klebsiella* were prevalent, with ESBL rates of 21.4% and 38.7%, respectively. These phenotypes are commonly driven by plasmid-borne CTX-M β-lactamases, which preferentially hydrolyze third-generation cephalosporins and increasingly circulate in community populations [21,22,23]. The high fluoroquinolone resistance observed in these species likely reflects decades of widespread outpatient use and the rapid accumulation of mutations in the quinolone resistance–determining regions (QRDRs) of gyrA and parC, often accompanied by plasmid-mediated qnr genes. Fluoroquinolone resistance is also associated with reduced expression of porins (such as OmpF in *E. coli*), further limiting antibiotic penetration and enhancing survival in hostile urinary environments [24,25].

The detection of OXA-48-like carbapenemase-producing *Klebsiella* isolates, although rare, is microbiologically significant. OXA-48 enzymes confer low-level carbapenem hydrolysis while preserving susceptibility to extended-spectrum cephalosporins, a phenotype that complicates laboratory detection and can facilitate silent dissemination [26]. Their presence suggests that carbapenem-resistant Enterobacterales (CRE) have entered local ecological networks, likely through patient transfer or environmental persistence, and may expand under continued selection pressure [26].

Polymicrobial infections in catheterized or immobile elderly women highlight the role of biofilm communities as reservoirs for genetic exchange and antimicrobial tolerance [27]. Mixed infections often included organisms such as *Pseudomonas aeruginosa*, whose quorum sensing systems, efflux pumps (MexAB-OprM), and type III secretion apparatus enable survival in complex microbial communities and enhance pathogenic potential [28]. The co-occurrence of *Candida* in a minority of cases further illustrates how biofilm-based interactions can modify antibiotic responsiveness, as fungal species are known to shield bacteria from immune clearance and alter local antibiotic gradients [29,30].

The logistic regression findings, identifying catheterization, recurrent infection, postmenopausal status, and prior antibiotic exposure as independent predictors of MDR infection, reflect the mechanistic underpinnings of microbial selection. Recurrent UTIs create iterative selection cycles that enrich for pathogens with enhanced adherence capacity, increased mutational adaptability, and plasmid exchanges within biofilm environments [31].

Antibiotic exposure disrupts protective commensals and favors the emergence of resistant clones, particularly in settings where horizontal gene transfer is facilitated by plasmids, integrons, and transposons [31,32].

Catheters introduce synthetic surfaces that select for biofilm-forming and inherently more resistant organisms, fundamentally altering the urinary tract’s microbial ecology [32].

Delayed adjustment from empiric to targeted therapy, largely due to MDR pathogens, underscores the clinical impact of these mechanistic processes [33]. As resistant organisms proliferate, their virulence traits—from siderophore production to immune evasion—amplify the risk of complications such as acute kidney injury and bacteremia, both of which were more prevalent in the oldest and most comorbid patients [34].

Prolonged hospitalization, observed in more than one-third of patients, reflects not only clinical severity but also the microbiological complexity and difficulty achieving rapid pathogen eradication in an environment shaped by resistance and impaired host defenses.

## 5. Conclusions

This study highlights the distinct clinical and microbiological profile of urinary tract infections in hospitalized women, particularly among postmenopausal patients. Age-related physiological changes, comorbidity burden, and increased healthcare exposure contributed to a shift from predominantly *E. coli* infections toward more opportunistic and healthcare-associated pathogens, including *Klebsiella* and *Enterococcus*. These organisms exhibited substantial antimicrobial resistance, with high rates of ESBL production and fluoroquinolone resistance, reflecting the selective pressures imposed by recurrent antibiotic use and prior hospitalization.

Multidrug-resistant infections were strongly associated with postmenopausal status, catheter use, and recent antimicrobial exposure, emphasizing the importance of targeted preventive strategies and judicious antibiotic stewardship in high-risk subgroups. Delays in appropriate therapy and the need for escalation in MDR cases resulted in prolonged hospitalization and greater clinical complexity.

Overall, the findings underscore the need for age-tailored management strategies, improved diagnostic precision, and enhanced surveillance of resistance trends to optimize care and reduce the burden of complicated UTIs in hospitalized women.

## Figures and Tables

**Table 1:** Comorbidities in 948 hospitalized adult women with symptomatic UTI.

Parameter	Total UTI n (%) N = 948
Chronic renal disease	237 (25)
Hypertension	587 (61.9)
Urinary tract obstruction	65 (6.8)
Nephrolithiasis	189 (19.9)
Malignancies	131 (13.8)
Heart failure	101 (10.6)
Stroke	154 (16.2)
Diabetes mellitus	206 (21.7)
HIV	52 (5.4)
Malnutrition	115 (12.1)
Cardiovascular disease	241 (25.4)
Hypertrophic prostate disease	146 (15.4)
Hospitalization 30 days prior	301 (31.7)

**Table 2:** Admission clinical and laboratory characteristics in the overall cohort. Values are n (%). Sepsis defined by Sepsis-3 criteria.

Parameter	Total UTI n (%) N = 948
Tachycardia	480 (50.6)
Fever	798 (84.1)
Sepsis	165 (17.4)
Haematuria	411 (43.3)
Elevated CRP	841 (88.7)
Leukocytosis	791 (83.4)
Hypotension	452 (47.6)
Acute kidney injury	272 (28.6)
Positive urine nitrites	305 (32.1)
Leukocyturia	832 (87.7)
Neutrophilia	696 (73.4)

**Table 3:** Baseline Demographic and Clinical Characteristics of the Study Population.

Characteristic	Total (n = 948)	Postmenopausal (n = 743)	Younger Women (n = 205)	*p*-Value
Age, median (IQR)	67 (58–78)	73 (66–82)	41 (34–47)	<0.001
Recurrent UTI (%)	37.9	42.6	18.3	<0.001
Diabetes mellitus (%)	28.6	34.1	10.8	<0.001
Chronic kidney disease (%)	19.3	22.4	6.5	<0.001
Frailty/limited mobility (%)	25.7	31.3	4.1	<0.001
Urinary incontinence (%)	23.9	28.5	6.9	<0.001
Indwelling urinary catheter (%)	14.7	17.6	3.7	<0.001
Prior antibiotic use (90 days) (%)	34.2	39.8	11.1	<0.001
Recent hospitalization (%)	24.4	28.7	8.5	<0.001
Acute kidney injury at admission (%)	8.9	10.2	3.9	0.004

**Table 4:** Distribution of uropathogens isolated from urine cultures in hospitalized adult female patients with symptomatic urinary tract infections, stratified by age group. Values are presented as number (percentage). Polymicrobial infection was defined as the isolation of two or more clinically significant uropathogens from the same urine culture.

Pathogen	Total Cases (n)	Percentage (% of 948)	Postmenopausal (%)	Younger Women (%)	*p*-Value
*Escherichia* *coli*	499	52.6	50.1	64.7	<0.01
*Klebsiella* spp.	174	18.4	20.3	12.8	0.01
*Enterococcus* spp.	115	12.1	14.8	4.9	<0.001
*Proteus* spp.	74	7.8	8.3	6.1	0.19
*Pseudomonas aeruginosa*	44	4.6	5.1	2.4	0.11
Other Gram-negative bacteria	27	2.9	3.0	2.4	0.68
*Candida* spp.	15	1.6	1.8	0.9	0.32
Polymicrobial infection	56	5.9	4.9	0.7	0.14

**Table 5:** Antimicrobial resistance rates among the main uropathogens according to EUCAST 2024. ESBL defined by combined-disk confirmation.

Pathogen	Antibiotic	Resistance (%)
***E. coli* (n = 499)**	Fluoroquinolones	34.9
	TMP-SMX	38.7
	ESBL phenotype	21.4
	Nitrofurantoin	5.8
***Klebsiella *** **spp. (n = 174)**	Fluoroquinolones	46.1
	Piperacillin–tazobactam	22.4
	ESBL phenotype	38.7
	Carbapenems	2.8
***Enterococcus *** **spp. (n = 115)**	High-level aminoglycosides	17.4
	Vancomycin	3.4
	Linezolid	0

**Table 6:** Multivariate analysis of independent predictors of MDR urinary tract infection.

Variable	Adjusted OR	95% CI	*p*-Value
Postmenopausal status	2.41	1.08–5.37	0.03
Recent antibiotic use (≤90 days)	3.62	1.95–6.73	<0.001
Urinary catheterization	4.12	2.01–8.44	<0.001
Recurrent UTI	2.89	1.41–5.95	0.004
Diabetes mellitus	1.47	0.76–2.82	0.21
Recent hospitalization	1.88	0.93–3.80	0.08

**Table 7:** Empiric and Targeted Antibiotic Therapy.

Variable	Total (%)	Postmenopausal (%)	Younger Women (%)	*p*-Value
Empiric therapy within 24 h	92.1	93.4	87.2	0.04
Empiric ceftriaxone therapy	53.8	57.3	39.1	0.01
Empiric fluoroquinolone therapy	21.5	19.8	28.1	0.07
Therapy modification required	27.8	30.4	18.0	0.003
Escalation due to MDR/ESBL	9.9	12.1	3.4	0.002
De-escalation after results	17.9	16.4	22.0	0.11
Time to targeted therapy (median hours)	48	52	36	<0.001

**Table 8:** Clinical Outcomes.

Outcome	Total (%)	Postmenopausal (%)	Younger Women (%)	*p*-Value
Length of stay, median (days)	6 (IQR 4–9)	7 (IQR 5–10)	4 (IQR 3–6)	<0.001
Prolonged hospitalization (>7 days)	32.4	37.9	13.7	<0.001
Acute kidney injury	8.9	10.2	3.9	0.004
Sepsis	5.1	5.8	2.4	0.12
Bacteremia	3.8	4.2	2.0	0.21
Catheter-related complications	11.2	13.1	3.9	0.001

## Data Availability

The original contributions presented in this study are included in the article. Further inquiries can be directed to the corresponding author.

## References

[1] Naghavi M., Vollset S.E., Ikuta K.S., Swetschinski L.R., Gray A.P., Wool E.E., Aguilar G.R., Mestrovic T., Smith G., Han C. (2024). Global burden of bacterial antimicrobial resistance 1990–2021: A systematic analysis with forecasts to 2050. Lancet.

[2] Paul M., Carrara E., Retamar P., Tängdén T., Bitterman R., Bonomo R.A., de Waele J., Daikos G.L., Akova M., Harbarth S. (2022). European Society of Clinical Microbiology and Infectious Diseases (ESCMID) guidelines for the treatment of infections caused by multidrug-resistant Gram-negative bacilli (endorsed by European society of intensive care medicine). Clin. Microbiol. Infect..

[3] Silva A., Costa E., Freitas A., Almeida A. (2022). Revisiting the Frequency and Antimicrobial Resistance Patterns of Bacteria Implicated in Community Urinary Tract Infections. Antibiotics.

[4] Gajdács M., Ábrók M., Lázár A., Burián K. (2021). Urinary Tract Infections in Elderly Patients: A 10-Year Study on Their Epidemiology and Antibiotic Resistance Based on the WHO Access, Watch, Reserve (AWaRe) Classification. Antibiotics.

[5] Esteve-Palau E. (2018). Impact of an Antimicrobial Stewardship Program on Urinary Tract Infections Caused by ESBL-Producing Escherichia coli. Rev. Esp. Quimioter..

[6] Polse R.F., Qarani S.M., Assafi M.S., Sabaly N., Ali F. (2020). Incidence and Antibiotic Sensitivity of Klebsiella pneumonia isolated from urinary tract infection patients in Zakho emergency hospital/Iraq. J. Educ. Sci..

[7] Muhammad A., Khan S.N., Ali N., Rehman M.U., Ali I. (2020). Prevalence and antibiotic susceptibility pattern of uropathogens in outpatients at a tertiary care hospital. New Microbes New Infect..

[8] Lorusso A.B., Carrara J.A., Barroso C.D.N., Tuon F.F., Faoro H. (2022). Role of Efflux Pumps on Antimicrobial Resistance in Pseudomonas aeruginosa. Int. J. Mol. Sci..

[9] Ballesteros-Monrreal M.G., Mendez-Pfeiffer P., Barrios-Villa E., Arenas-Hernández M.M.P., Enciso-Martínez Y., Sepúlveda-Moreno C.O., Bolado-Martínez E., Valencia D. (2023). Uropathogenic Escherichia coli in Mexico, an overview of virulence and resistance determinants: Systematic review and meta-analysis. Arch. Med. Res..

[10] Faine B.A., Rech M.A., Vakkalanka P., Gross A., Brown C., Harding S.J., Slocum G., Zimmerman D., Zepeski A., Rewitzer S. (2022). High prevalence of fluoroquinolone-resistant UTI among US emergency department patients diagnosed with urinary tract infection, 2018–2020. Acad. Emerg. Med..

[11] Rodriguez-Mañas L. (2020). Urinary tract infections in the elderly: A review of disease characteristics and current treatment options. Drugs Context.

[12] Al-Orphaly M., Hadi H.A., Eltayeb F.K., Al-Hail H., Samuel B.G., Sultan A.A., Skariah S. (2021). Epidemiology of Multidrug-Resistant Pseudomonas aeruginosa in the Middle East and North Africa Region. mSphere.

[13] Huang L., Huang C., Yan Y., Sun L., Li H. (2022). Urinary tract infection etiological profiles and antibiotic resistance patterns varied among different age categories: A retrospective study from a tertiary general hospital during a 12-year period. Front. Microbiol..

[14] Kraszewska Z., Skowron K., Kwiecinska-Piróg J., Grudlewska-Buda K., Przekwas J., Wiktorczyk-Kapischke N., Wałecka-Zacharska E., Gospodarek-Komkowska E. (2022). Antibiotic resistance of Enterococcus spp. isolated from the urine of patients hospitalized in the university hospital in North-Central Poland, 2016–2021. Antibiotics.

[15] Khalil M.A., Alorabi J.A., Al-Otaibi L.M., Ali S.S., Elsilk S.E. (2023). Antibiotic resistance and biofilm formation in Enterococcus spp. isolated from urinary tract infections. Pathogens.

[16] Ismail F., Haq S., Aiayad M., Abushiba M., Zorgani A. (2022). Antibiotic resistance patterns of urinary pathogens in outpatients and inpatients: A report from Eastern Libya. Int. J. Urol. Nurs..

[17] Patel J., Harant A., Fernandes G. (2023). Measuring the global response to antimicrobial resistance, 2020–2021: A systematic governance analysis of 114 countries. Lancet Infect. Dis..

[18] Lewnard J.A., Charani E., Gleason A. (2024). Burden of bacterial antimicrobial resistance in low-income and middle-income countries avertible by existing interventions: An evidence review and modelling analysis. Lancet.

[19] Popovich K.J., Aureden K., Ham D.C. (2023). SHEA/IDSA/APIC practice recommendation: Strategies to prevent methicillin-resistant Staphylococcus aureus transmission and infection in acute-care hospitals: 2022 update. Infect. Control Hosp. Epidemiol..

[20] Laxminarayan R., Impalli I., Rangarajan R. (2024). Expanding antibiotic, vaccine, and diagnostics development and access to tackle antimicrobial resistance. Lancet.

[21] Sabbatucci M., Ashiru-Oredope D., Barbier L. (2024). Tracking progress on antimicrobial resistance by the quadripartite country self-assessment survey (TrACSS) in G7 countries, 2017–2023: Opportunities and gaps. Pharmacol. Res..

[22] Geerlings S.E. (2021). Genital and Urinary Tract Infections in Diabetes: Impact of Glucosuria. Diabetes Res. Clin. Pract..

[23] Nitzan O. (2020). UTIs in Type 2 Diabetes: Prevalence, Diagnosis, and Management. Diabetes Metab. Syndr. Obes..

[24] Veeraraghavan B., Bakthavatchalam Y.D., Sahni R.D. (2021). Oral Antibiotics in Development for Community-Acquired UTIs. Infect. Dis. Ther..

[25] Mohanty S. (2022). Diabetes Downregulates Psoriasin and Increases *E. coli* Burden in the Bladder. Nat. Commun..

[26] Bader M.S., Loeb M., Leto D., Brooks A.A. (2020). Treatment of UTIs in the Era of Resistance and New Agents. Postgrad. Med..

[27] Li X., Fan H., Zi H. (2022). Global and Regional Burden of Antimicrobial Resistance in UTIs (2019). J. Clin. Med..

[28] Bonomo R.A., Perez F., Hujer A.M., Hujer K.M., Vila A.J. (2024). The real crisis in antimicrobial resistance: Failure to anticipate and respond. Clin. Infect. Dis..

[29] Naber K.G., Wagenlehner F., Kresken M. (2023). *Escherichia coli* resistance, treatment patterns and clinical outcomes among females with uUTI in Germany: A retrospective physician-based chart review study. Sci. Rep..

[30] Ormeño M.A., Ormeño M.J., Quispe A.M. (2022). Recurrence of urinary tract infections due to *Escherichia coli* and its association with antimicrobial resistance. Microb. Drug Resist..

[31] Behzadi P. (2020). Classical Chaperone-Usher Adhesive Fimbriome of UPEC in UTIs. Folia Microbiol..

[32] Helmi R.T., Al-Maqbali J.S., Gamal S., Ba Wazir H., Al Sulemani Y., Al Za’abi M. (2024). Short-term effects of antimicrobial stewardship programs on antibiotics usage, clinical outcomes, and multidrug resistant organisms in the post COVID-19 era. J. Infect. Public Health.

[33] Effah C.Y., Sun T., Liu S., Wu Y. (2020). Klebsiella pneumoniae: An Increasing Threat to Public Health. Ann. Clin. Microbiol. Antimicrob..

[34] Qin S. (2022). Pseudomonas aeruginosa: Pathogenesis, Virulence, and Emerging Therapeutics. Signal Transduct. Target. Ther..

